# Caregiving, Loneliness, and Stress: The Role of COVID-19

**DOI:** 10.1093/geroni/igab046.3671

**Published:** 2021-12-17

**Authors:** Sumaiyah Syed, Iris Yang, Stephanie Wilson

**Affiliations:** 1 Southern Methodist University, Dallas, Texas, United States; 2 Southern Methodist University, DALLAS, Texas, United States

## Abstract

According to many prior studies, informal caregivers are at risk for heightened loneliness and distress. Moreover, the COVID-19 pandemic has introduced new challenges that may be accentuated among caregivers. This study examined caregiving frequency and its relation to loneliness, perceived stress, and negative affect during the COVID-19 pandemic. We then investigated the moderating roles of COVID-19 concerns and impacts, anticipating magnified effects among caregivers. Participants included respondents from the Health and Retirement Study (HRS) COVID-19 project sample (n=2108, mean age=69) who reported their caregiving frequency, general concern about COVID-19, related concerns about family members’ health, and social disruptions caused by the pandemic. Controlling for age, gender, and health status, daily caregivers reported significantly greater distress during COVID-19 compared to non-caregivers (p=.036). Higher levels of concern about family members’ health during COVID-19 was significantly associated with greater loneliness among daily caregivers (p = .009), but not among non-caregivers, such that daily caregivers with greater concerns had the highest levels of loneliness. On the other hand, unexpectedly, daily caregivers who experienced fewer social disruptions due to the pandemic reported higher levels of loneliness (p = .002); however, the association was null for non-caregivers. Findings suggest that daily caregivers may be particularly vulnerable to greater loneliness and stress during the COVID-19 pandemic. These experiences may exacerbate existing mental health disparities for those providing daily care.

